# Distal humerus fracture in patients over 70 years of age: results of open reduction and internal fixation

**DOI:** 10.1007/s00402-020-03664-4

**Published:** 2020-11-05

**Authors:** Mohamed Moursy, Kilian Wegmann, Florian Wichlas, Mark Tauber

**Affiliations:** 1 Department of Orthopedics and Traumatology, Paracelsus Medical University, Salzburg, Austria; 2 University of Cologne, Faculty of Medicine, Cologne, Germany; 3 University Hospital Cologne, Center of Orthopedic and Trauma Surgery, Cologne, Germany; 4 Shoulder and Elbow Surgery, ATOS Clinic, Munich, Germany

**Keywords:** Distal humerus fracture, Geriatric trauma, Locked compression plate, Trauma

## Abstract

**Background:**

Due to the complexity of distal humerusfractures and often poor bone quality in elderly patients, these entities remain a challenge. However, because of a high rate of complications related to total elbow prostheses, reconstruction of distal humerus fractures should still be considered a therapeutic option, also in the elderly patient. The purpose of the present study was to investigate the clinical outcomes after open reduction and internal fixation and to evaluate whether the results justify reconstruction even in elderly patients. We hypothesized that despite advanced age, reasonable clinical results can be achieved, using a standardized surgical technique and aftertreatment protocol for the treatment of distal humerus fractures in elderly patients.

**Methods:**

Between 2004 and 2012, 30 patients with a mean age of 78 years at the time of injury with a recent distal humerus fracture were evaluated. All patients underwent the identical aftertreatment protocol with no weight bearing for 6 weeks and weekly increasing range of motion. Follow-up rate was 90%. 22 patients were treated with double plate, 4 with single plate, and 1 with screw fixation only. Patients were evaluated based on clinical criteria. Primary outcome measures were Mayo Elbow Performance Score, VAS and joint range of motion, secondary was radiological evaluation.

**Results:**

After a mean follow-up period of 3.8 years (min. 1 year, max. 9 years, SD ± 2), the average range of motion was flexion of 127° (min. 100°; max. 150°; SD ± 16.5) and average loss of extension of 20.9° (min. 5°; max. 40°; SD ± 11). Average pronation and supination was 68.3° (min. 0°; max. 90°; SD ± 25.3) and 75.3° (min. 0°; max. 90°; SD ± 19.7), respectively. Average Mayo Elbow Performance (MEPS) score was 88.7 (min. 60; max. 100; SD ± 12.1). 6 patients developed heterotopic ossification without significant effect on the clinical outcome. 7 patients had radiological evidence of at least partial non-union with one requiring revision, 2 discrete hardware dislocations were treated conservatively. There were no infections in the presented cohort. Our results regarding the surgical approach showed significantly higher patient satisfaction scores in the osteotomy group, compared to the group with Triceps-On Approach (PTOA).

**Conclusion:**

The present data support indication for open reduction internal fixation (ORIF) even in the elderly patient. Advanced age should not be seen as a contraindication for ORIF of fractures of the distal humerus. Although the rate of complications is higher than in younger patients, complications such as non-union are often asymptomatic, patient satisfaction scores are high, and the possible devastating complications of failed elbow replacement can be evaded.

**Level of evidence:**

IV.

## Introduction

Distal humeral fractures in the elderly population are on the rise and only little literature exists regarding the fixation. With an increasingly aging population in most countries in the western world, it is of value to know the likely outcome of fracture fixation of the distal humerus in this age group. Court-Brown and Caesar reported on the development of fracture patterns in a stable population of around 500,000 people around Edinburgh [1]. They reported an incidence of distal humeral fractures of 5.8 per 100,000 and noted that this was only 0.5% of all fractures seen. Interestingly, the distribution of distal humerus fractures showed a ‘unimodal older woman’ pattern and falls in what they describe as an osteoporotic curve pattern. This type of curve pattern was not seen in the previous study of Buhr and Cooke from 1959 suggesting that these fractures are on the rise [2]. The largest epidemiological study in the world comes from the Finnish National Hospital Discharge Register; this study looked at all distal humeral fractures in women over the age of 60 for the years 1970–1995. The most significant increase was seen in the oldest women, with an age specific increase of 8 in 1970 and 54 in 1995, representing a sevenfold increase [3]. For all women over 60 years the incidence of distal humerus fractures will triple over the next 20 years [4]. Only a few studies concerning the fixation of these fractures in patients over the age of 70 years were published in the last decade. Studies with large numbers and sufficient follow-up are understandably difficult to achieve in this study population, so any further information is valuable. While these fractures are uncommon, representing only 1–2% of fractures occurring in adults, they are difficult to treat, with options ranging from non-operative management to total elbow replacement [5, 6]. According to the recent literature, total elbow replacement (TEA) comes with a high rate of complications like loosening, deep infection, ulnar nerve lesions. Depending on linked and unlinked systems, the complication rate has been reported to be at 19 and 26% [7, 8]. Assuming an increase of this type of injury and the workload that it will represent in the future, we looked at the functional and radiological outcomes and complications of all our patients aged 70 years and older treated with ORIF for distal humerus fractures over a period of 8 years, to evaluate whether ORIF can achieve adequate results even in elderly patients.

## Patients and methods

**Fig. 1:**
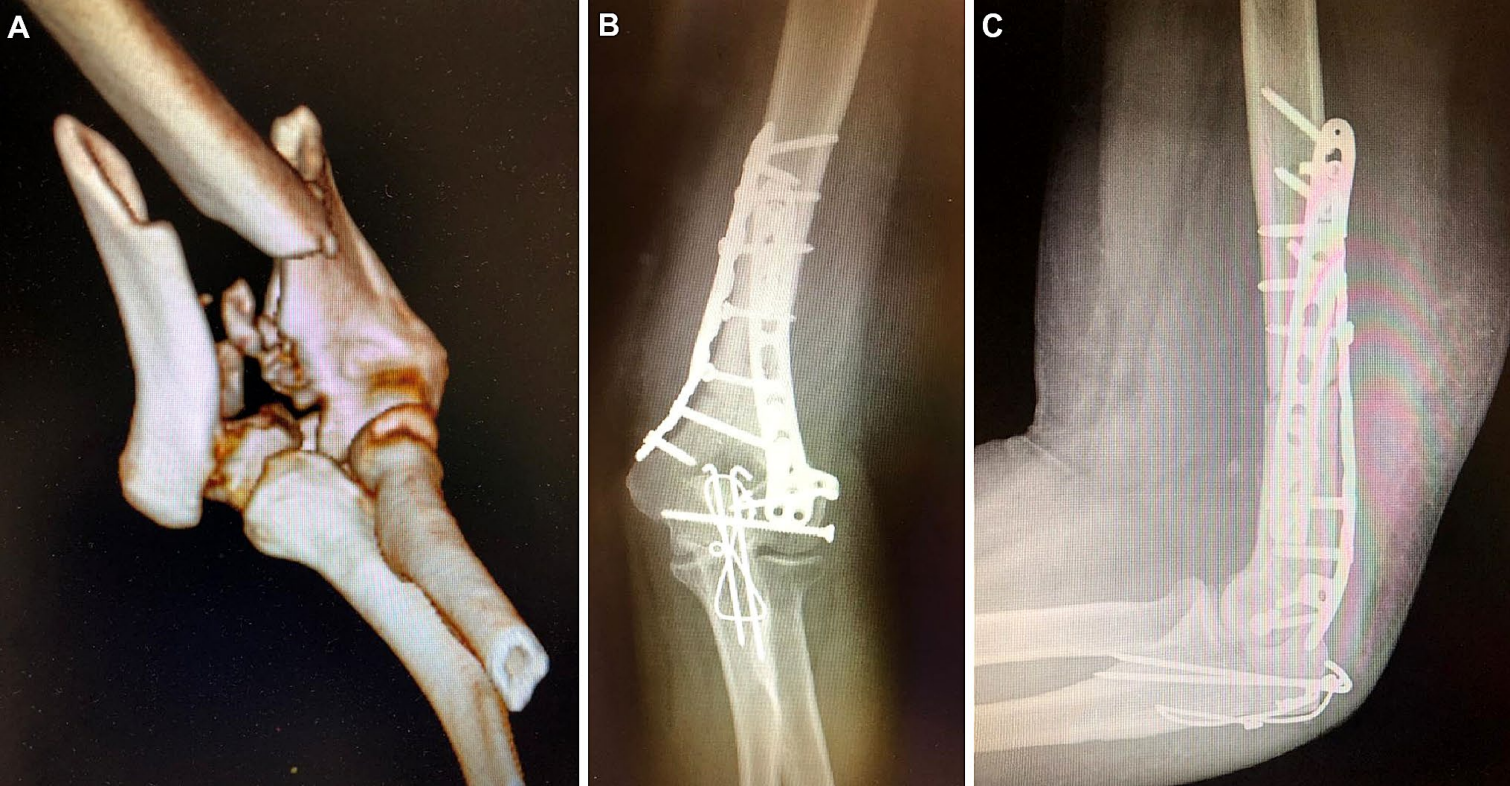
**a** Initial 3D CT-scan of a 71-year-old female patient, having suffered an AO 13 C3 fracture of her left arm by a fall from standing height. **b**, **c** Final follow-up X-rays after 2 years with stable fixation and bony healing

All patients with distal humeral fractures operatively treated with ORIF over an 8-year period between 2004 and 2012 at an urban level 1 trauma centre were retrospectively reviewed. Written informed consent of the patients and approval by the local ethics committee were achieved. The cohort consisted of 30 patients, (27 women and 3 men). Mean age at time of injury was 78 years (range 70–90, SD ± 5.3). 3 patients were lost to follow-up due to natural death, resulting in a 90% follow-up rate for our collective (27 patients). Of these injuries, 6 were Type A, 5 Type B and 16 Type C as per AO Classification (Table 1). Five patients had suffered open fractures (3 Type I and 2 Type II) according to Gustilo–Anderson classification [9, 10]. Average follow-up time was 3.84 years (range 1.1–8.8, SD ± 2), with a minimum of 1 year. 22 patients were treated with double plate osteosynthesis, 4 with a single plate and 1 with screw fixation only. All patients received a plaster of Paris cast post operatively for an average of 2.2 weeks (range 2–6) until removal of skin sutures. The surgical approach involved a posterior midline incision with olecranon osteotomy (*n* = 11) of chevron type, a posterior triceps-on approach (PTOA) according to Alonso-Llamas, where the distal humerus is approached medially and laterally avoiding detachment of the triceps and avoiding osteotomy of the olecranon (*n* = 14), muscle splitting (*n* = 1) or lateral approach only (*n* = 1) (Table 2) [11]. The osteotomized olecrani were all fixed with a tension band wire technique. In 19 out of 27 patients who showed initially post-traumatic neuropathy of the ulnar nerve or in those, where the nerve was irritated by the positioned osteosynthetic implants, anterior transposition of the ulnar nerve was performed at the time of surgery. Aftertreatment protocol was set identical in all patients with no weight bearing for 6 weeks and then gradual increase of loads after 6-week X-ray control. The range of motion was limited to extension/flexion of 0°/30°/90° for the first week, with then increasing range of motion under physiotherapy over the following weeks. Due to the age of the cohort, not all patients were able to strictly adhere to the protocol. At the final follow-up patients were evaluated clinically and radiologically by an independent observer and X-ray imaging in two orthogonal planes (a.p. and lateral view). Follow-up was at 2, 6, 12 weeks, 6 months and minimum 1 year. Outcome criteria were range of motion, pain [visual analogue scale (VAS)] with 0 meaning no pain and 10 maximum pain), satisfaction score (VAS with 0 meaning unsatisfied and 10 very satisfied) and Mayo elbow performance score (MEPS) with a score of 100 being optimal result. Primary outcome measures were Mayo Elbow Performance Score, VAS and joint range of motion, secondary was radiological evaluation (Fig. 1).

**Table 1:** Fracture types according to AO classification

Fracture type	Number of cases
A1	0
A2	5
A3	1
B1	2
B2	2
B3	1
C1	4
C2	3
C3	9

**Table 2:** Patient data

Age at injury	AO classification of fractures	Approach	Fixation method	Ulnar nerve transpo-sition
76	B1, 3	Lateral	Screws only	No
78	C1, 2	OO	1 radial plate	No
78	B1, 1	PTOA	1 radial plate	No
74	A2, 3	PTOA	2 plates	Yes
76	A2, 3	PTOA	2 plates	Yes
71	C3, 2	OO	2 plates	Yes
80	A3, 2	PTOA	2 plates	Yes
85	C1, 2	OO	2 plates	Yes
71	C1, 2	OO	2 plates	Yes
74	C2, 2	OO	2 plates	Yes
70	C1, 1	PTOA	1 radial plate	No
82	C3, 3	OO	2 plates	Yes
83	C3, 3	OO	2 plates	Yes
77	C3, 3	OO	2 plates	Yes
76	C2, 2	OO	2 plates	Yes
72	C3, 2	Muscle splitting	2 plates	Yes
76	A2, 2	PTOA	2 plates	Yes
76	B3, 3	PTOA	1 radial plate	No
71	A2, 3	PTOA	2 plates	Yes
87	B2, 3	PTOA	2 plates	No
79	A2, 3	PTOA	2 plates	No
90	C2, 3	OO	2 plates	Yes
78	C3, 2	OO	2 plates	Yes
79	C3, 3	PTOA	2 plates	Yes
85	C3, 2	PTOA	2 plates	Yes
86	C3, 1	PTOA	2 plates	Yes
80	B2, 3	PTOA	2 plates	No

*PTOA *posterior triceps-on approach, *OO *olecranon osteotomy

Statistical analysis between the osteotomy group and the PTOA group was done with STATISTICA 6.0 (StatSoft, Inc. STATISTICA (data analysis software system). The clinical results in both groups were compared using the Mann–Whitney *U* test. A *p* value less than 5% was considered statistically significant. Effect size (*R*) was calculated as the quotient of the z value and the square root of the sample size.

## Results

**Fig. 2:**
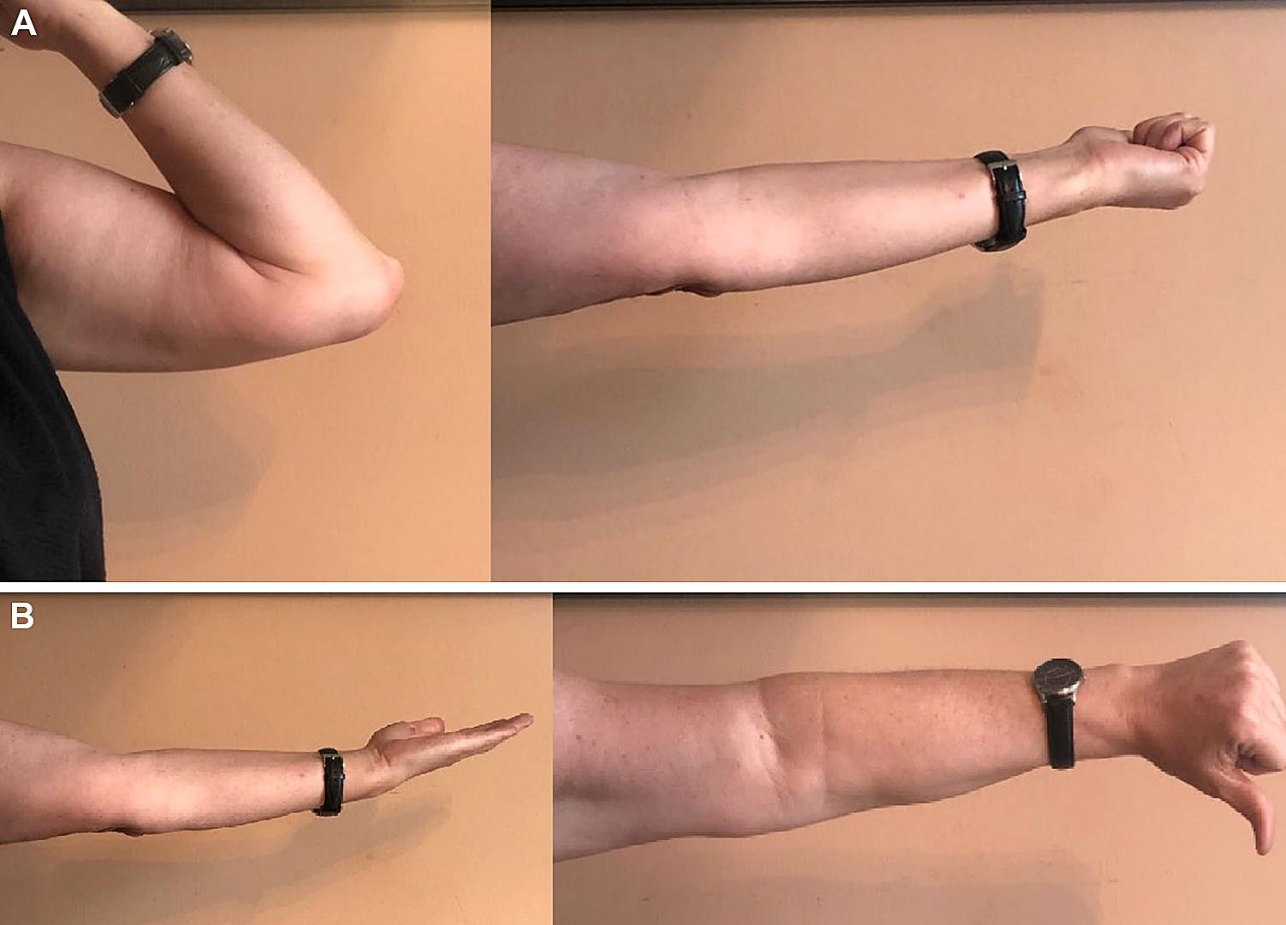
Final clinical follow-up after 2 years with slight extension deficit of 10°, but free flexion and rotation

Mean follow-up period of 3.8 years (min. 1 year, max. 9 years, SD ± 2) the average range of motion was flexion of 127° (min. 100°; max. 150°; SD ± 16.5) and average loss of extension of 20.9° (min. 5°; max. 40°; SD ± 11). Average pronation and supination was 68.3° (min. 0°; max. 90°; SD ± 25.3) and 75.3° (min. 0°; max. 90°; SD ± 19.7), respectively (Fig. 2).

Average pain score was 1.3 out of 10 (range 0–7, SD ± 1.7) and patient satisfaction score was 8.8 (range 5–10, SD ± 1.4). MEPS averaged 88.7 (range 60–100, SD ± 12.1). The MEPS was excellent (90–100) in 17 patients (63%), good (75–89) in 6 patients (22%), fair (61–74) in 3 patients (11%) and poor (60 or less) in 1 patient (4%) (Table 3). The average MEPS for the unaffected elbow was 89 (range 90–100) by comparison. Six patients developed heterotopic ossification (22%), 5 of these 6 were in patients who had undergone olecranon osteotomy. There was no significant difference in the range of motion outcome between the group with heterotopic ossification (mean range − 20° extension to 123 degrees flexion) and without (mean range − 22° extension to 128 degrees flexion). Six patients mal-united, 4 of which in a valgus position (average 8 degrees) and 2 in a varus position (average 10°). Six patients (22%) had clinical and radiological evidence of non-union: three occurred in the olecranon osteotomy group and three in the PTOA group. One required revision plating at 4 months. Hardware complications were observed in two patients, one of whom underwent revision of olecranon fixation; the other one had a plate loosening which was treated conservatively. Two patients, both of whom had undergone ulnar nerve transposition, experienced transient ulnar nerve palsy; one had sustained neurapraxia at time of injury, the other one at time of surgery. Both resolved over time. There were no post-operative infections in the present cohort. Comparing patients with olecranon osteotomy and the ones with PTOA, we found no significant difference between range of motion, pain score and MEPS whereas for satisfaction score, the difference was statistically significant (*p* < 0.05) in favour of olecranon osteotomy (Table 4), despite the higher rate of complex intra-articular fractures treated with this approach. Effect size was found to be *r* = 0.6325, with *r* values above 0.5 being seen as strong effect.

**Table 3:** Clinical outcome

Follow-up (years/months)	Fracture type	Extension	Flexion	Functional arc of motion	Pronation	Supination	Pain score VAS	Satisfaction VAS	Mayo Elbow Score
6.2	B1, 3	5	140	135	80	80	0	10	100
5.6	C1, 2	20	150	130	70	0	0	10	95
6.1	B1, 1	10	120	110	70	90	2	9	85
1.4	A2, 3	30	100	70	0	30	2	10	70
1.7	A2, 3	20	150	130	20	80	5	8	75
2.2	C3, 2	40	100	60	85	90	0	10	90
2.8	A3, 2	30	120	90	85	90	2	7	95
1.1	C1, 2	20	100	80	0	80	3	10	75
3.5	C1, 2	20	135	115	90	90	0	10	100
5.6	C2, 2	20	135	115	90	90	1	9	100
3.8	C1, 1	10	150	140	80	80	0	10	100
4.1	C3, 3	10	110	100	80	80	3	9	85
3.9	C3, 3	10	150	140	40	80	0	10	100
5.6	C3, 3	10	140	130	50	80	0	10	100
8.8	C2, 2	10	150	140	80	80	0	10	100
1.3	C3, 2	5	130	125	80	80	0	10	100
5.1	A2, 2	40	140	100	70	70	2	7	80
5.3	B3, 3	40	100	60	70	80	3	7	70
1.2	A2, 3	30	120	90	90	80	7	5	60
3.1	B2, 3	30	110	80	60	50	0	7	90
2.7	A2, 3	30	130	100	80	80	1	9	100
4.2	C2, 3	20	120	100	90	90	1	9	80
3.8	C3, 2	20	120	100	70	70	1	9	95
3.7	C3, 3	10	130	120	80	80	1	8	90
4.1	C3, 2	40	120	80	70	70	0	7	70
5.5	C3, 1	15	130	115	80	80	0	9	100
1.3	B2, 3	20	130	110	85	85	2	8	90

**Table 4:** Comparison of outcomes with approach variation

Characteristics	Combined (*n* = 25)	OO (*n* = 11)	PTOA (*n* = 14)	*p*	Test
Mean (SD)					
Extension (negative)	22 ± 11	18 + /9	25 ± 0	0.09	T
Mean flexion	126 ± 17	128 ± 19	125 ± 16	0.65	T
Functional arc of motion	104 ± 24	110 ± 25	100 ± 23	0.29	T
Mayo Elbow Score	88 ± 12	93 ± 9	84 ± 13	0.07	T
Median (min–max)					
Pronation	80 (0–90)	80 (0–90)	75 (0–90)	0.66	W
Supination	80 (0–90)	80 (0–90)	80 (30–90)	0.36	W
Pain score VAS	1 (0–7)	0 (0–3)	2 (0–7)	0.12	W
Satisfaction VAS	9 (5–10)	10 (9–10)	8 (5–10)	0.001	W

*OO *olecranon osteotomy, *PTOA *posterior triceps-on approach, *VAS *visual analog scale, *T T* test, *W *Wilcoxon rank-sum test

## Discussion

Based on the present data, ORIF of distal humerus fractures still is a reliable treatment option for fractures of the distal humerus in the elderly. Our series found mostly excellent outcome scores with a low revision rate for patients over 70 years of age.

Several strategies exist for dealing with distal humerus fractures in the elderly, and decision-making depends on fracture configuration, but also patient characteristics. While simple fractures may be sufficiently fixed with only screws [12, 13], more complex patterns of type A fractures as well as type B and C fractures demand plate fixation or even prosthetic replacement. Clavert et al. concluded that for AO type A and B fractures, osteosynthetic fixation provided the best results if the patients had little medical history, no neuropsychiatric disorders and if the fracture occurred on the dominant side [14]. The main predictor of failure was osteoporosis, which increased the risk of clinical and radiological failure fourfold regardless of the type of construct or hardware used [14]. Relevant comorbidities that have significant impact on life expectancy will favour conservative treatment modalities, as shown by the overview of Lander and colleagues [15]. The authors found reasonable results by immobilisation of complex distal humerus fractures in elderly low-demand patients. But Lander’s paper also showed a reduction in mortality, if operative treatment was chosen. In their recent paper, Goyal et al. investigated the risk for re-operation of elderly patients after total elbow arthroplasty or osteosynthesis. Summarizing the results, patients after total elbow arthroplasty had a lower risk of re-operation and a higher death rate at the time of the follow-up, compared to patients after osteosynthesis. Interestingly, the authors found no difference in re-operation when looking at the time interval when angle-stable implants had been used for osteosynthesis. The data might implicate that with modern techniques, reconstruction is reliably possible even in elderly patients. The study of Goyal et al. shows that despite several other studies may have indicated that in elderly patients, arthroplasty should be preferred over osteosynthesis for distal humerus fractures [16–19], the topic is not closed for discussion. There is still a need for data on the outcomes of distal humerus reconstruction in elderly patients, to feed the discussion.

Although the information that reconstruction of distal humerus fractures is not new [20–22], the cohort of this study is of a reasonable size and the data in our eyes add to the body of literature. The present paper reports the experiences of the authors but does not offer precise decision-making guidance for the treatment of distal humerus fractures in elderly patients. It is nearly impossible to find clearly defined cut-off values for when to reconstruct or when to replace the distal humerus. But it documents reliable results for reconstruction. The advantage of reconstruction is retaining the native bone and articular structures. Retaining the bone prevents loosening or implant failure, as it is seen in patients with prosthetic replacement [23]. The few existing reports of internal fixation in general yield good results [24–28]. The guiding principle for the treatment of these fractures remains anatomical reduction at the level of articular cartilage and stable fixation at the level of the columns [29, 30]. The majority of the patients of the present study were treated with ORIF with double plates in an orthogonal pattern. Recent discussions focused on the configuration of double plates at the distal humerus, as a strict medio-lateral plate configuration in a 180° fashion yielded higher primary stability in in vitro study setups, compared to orthogonal plating in 90° [31, 32]. Yet, when looking at the impact of plate position on the clinical outcome, no significant differences could be found [33–36]. In a prospective randomised study Shin et.al observed no significant difference between the parallel plating method and the orthogonal plating with regard to non-union rates, range of motion and MEPS [36]. Good to excellent MEPSs were observed in 85% of our patients which confirms this statement. Our range of motion outcomes closely matches those seen in similar studies with an average population age of 72 years [37, 38]. The literature is not able to favour either total elbow arthroplasty or internal fixation [39]. Both are surgically challenging in this patient group. Achieving sufficient patient numbers for prospective randomized trials in this age group will almost certainly require multi-centre collaboration.

To the best of our knowledge, our series represents the largest patient cohort treated with ORIF in this age group as published in the literature. Pain scores were low and the complication rates were comparable to those seen in studies with a younger patient base. Our patients were not formally assessed in terms of their bone mineral density, but it is reasonable to assume that in a predominantly female population of 75, osteoporosis will likely be prevalent and indeed epidemiological studies suggest that distal humeral fractures in this age group should now be considered osteoporotic fractures [1]. When PTOA is used, the surgeon has the option to convert to joint replacement, if reconstruction is not feasible. In addition, revision of patients who underwent PTOA is easier to handle with TEA in comparison to those with OO. Thus, in our eyes, OO should be done only after the surgeon sees a realistic chance of stable fixation, especially in the elderly patients.

Our rate of heterotopic ossification at 22% is similar to that reported in other series [25, 40]. It is of note that the presence of heterotopic ossifications did not influence the functional outcome. Transposing the ulnar nerve made no difference to outcome and was predominantly used in cases where nerve mobilization facilitated the fixation. We do not transpose the nerve routinely.

Finally, several limitations of our study have to be mentioned. First, the retrospective study design has to be mentioned. However, a follow-up rate of 90% is more than acceptable for this group of patients and all clinical and radiological evaluations were completed. No comparison group is available, and all surgeries were performed at one single center. The overall number of patients seems to be moderate; but considering the low fracture incidence, this number seems to be acceptable for a single center when compared to other studies.

Successful ORIF in our eyes has potential advantages over joint replacement in the long term although the present study is not fit to prove advantages. ORIF should have place in contemporary treatment plans for distal humerus fractures of elderly patients. Nevertheless, the very distal fractures, or low fractures are difficult to be fixed and replacement should be chosen. This is even more the case when obvious signs of highly reduced bone quality are present. In those with a massive osteoporosis and comminuted low fractures, we consider total elbow arthroplasty as a reliable treatment modality. Hence, we believe that it is worth giving patients regardless of their age a chance to restore the anatomy if possible, and not to promote cut-off values in age for example, beyond which no reconstruction is undertaken anymore. In that regard, we should take into consideration the increasing level of demand in the aging population and should evaluate the “biological age” of the patient and not the absolute numbers.

## Conclusions

Outcomes of fractures were good or excellent in 85% of our patients according to their Mayo Elbow Performance Score. Pain scores were also low. A good functional result does of course not reflect the patients overall state of health. However, any treatment that can preserve independence in the elderly has to be valued. Advanced age should not be a contraindication to open reduction and fixation in distal humerus fractures.
